# Updating the Brazilian clinical practice guidelines for sickle cell disease: Recommendations and development process

**DOI:** 10.1016/j.htct.2025.103964

**Published:** 2025-08-23

**Authors:** Ludmila Peres Gargano, Mariana Millan Fachi, Layssa Andrade Oliveira, Clarisse Lobo, Katharina Nelly Tobos Melnikoff, Selma Soriano, Marta da Cunha Lobo Souto Maior, Meline Rossetto Kron-Rodrigues, Dalila Fernandes Gomes, Haliton Alves Oliveira Junior, Rosa Camila Lucchetta

**Affiliations:** a Hospital Alemão Oswaldo Cruz, São Paulo, Brazil; b Instituto Estadual de Hematologia Arthur de Siqueira Cavalcanti, SES, Rio de Janeiro, Brazil; c Coordenadoria de Saúde Sudeste do Município de São Paulo, São Paulo, Brazil; d Brazilian Ministry of Health's (MoH), Brasília, Brazil

**Keywords:** Sickle cell anemial, Practice guidelines, Public health, Health technology assessment

## Abstract

**Background:**

Sickle cell disease is a hereditary blood disorder that significantly impacts morbidity and mortality, requiring comprehensive care. In Brazil, its management in the National Health Service follows the Brazilian Clinical Practice Guidelines, based on evidence and expert consensus. Periodic updates ensure alignment with new scientific findings.

**Objectives:**

This study describes the methodology for updating the clinical guidelines for sickle cell disease and provides an overview of recommendations for diagnosis, treatment and monitoring, emphasizing the evidence and health technology assessments for prioritized technologies.

**Methods:**

The update followed the technical guide of the Brazilian Ministry of Health, and the Gradings of Recommendation, Assessment, Development and Evaluation (GRADE) approach. All the recommendations were assessed by the National Committee for Health Technology Incorporation (Conitec). The clinical guidelines panel included health technology assessment researchers, clinical experts, and policymakers. Systematic reviews assessed new evidence with stakeholder contributions being incorporated through public consultation. Cost-effectiveness analysis was applied to support new technology coverage or changes.

**Results:**

The updated clinical guidelines provide structured recommendations for screening, diagnosis, prophylaxis, vaccination, and treatment, covering pharmacological and non-pharmacological approaches. It emphasizes patient and caregiver education to promote early recognition of complications. Expected benefits include fewer pain crises, fewer hospitalizations and transfusions, and improved fetal hemoglobin level, quality of life and survival rates. Key updates include listing epoetin alfa and 100 mg hydroxyurea tablets, expanding hydroxyurea eligibility criteria and revising monitoring protocols.

**Conclusion:**

The updated clinical practice guidelines standardize sickle cell disease care in the Brazilian NHS aligned with current evidence. Dissemination and integration aim to enhance healthcare delivery, while future assessments should optimize real-world implementation.

## Introduction

Sickle Cell Disease (SCD) is a genetic condition encompassing a group of hereditary disorders caused by alterations in the structure of hemoglobin (Hb) that may be associated with defects in its synthesis [1,2]. Sickle-shaped red blood cells (RBCs) are rigid and exhibit membrane alterations, making them prone to rupture, leading to intravascular hemolysis and chronic hemolytic anemia. Additionally, sickled RBCs express an increased quantity of adhesion molecules, promoting heightened interactions with endothelial cells, leukocytes, and platelets [1,2] These processes, coupled with endothelial damage, proliferative vasculopathy, and hypercoagulability, contribute to the primary clinical manifestations of SCD, including vaso-occlusive episodes, acute pain, inflammatory responses, and multi-organ damage [3, 4, 5, 6].

SCD is among the most common genetic disorders worldwide with an estimated 5 % of the global population carrying the beta-S globin mutation [7]. Hemoglobinopathies resulting from structural hemoglobin defects are particularly prevalent in African populations, where approximately 15 % of infant mortality is attributed to SCD [8]. This elevated prevalence of the Hb S gene among populations of African descent is reflected globally [9].

In Brazil, approximately 4 % of the population carries the sickle cell trait, with a heterogeneous distribution across regions [10,11]. According to the annual data report of the National Neonatal Screening Program, 5428 newborns were diagnosed with SCD between 2014 and 2018 [12]. While recent data are lacking, estimates from 2007 suggest that 25,000 to 50,000 individuals in Brazil are affected by sickle cell anemia (Hb SS homozygotes), compound heterozygosity, or double heterozygosity for SCD [11,13].

Due to extensive racial mixing and population migrations, SCD is present throughout the Brazilian territory. Although the condition is not exclusive to any single group and can occur in individuals of all skin tones, it is most prevalent among populations with African, Mediterranean, Middle Eastern, and South Asian ancestry [11]. The estimated prevalence of the sickle cell trait ranges from 6-10 %. Historically, sickle cell trait was introduced to Brazil via the transatlantic slave trade, as enslaved individuals were forcibly brought from Africa [14]. Consequently, SCD remained neglected for a long time, rendered invisible by structural and institutional racism [15,16].

Despite being a prevalent genetic disorder, described over a century ago, with significant health and economic burden [17,18], patients with SCD are particularly vulnerable to health care disparities and treatment options for SCD remain limited [19,20]. Hydroxyurea (HU), blood transfusions, and stem cell transplantation are currently the primary therapeutic approaches, but access and utilization vary greatly [21]. This underscores the urgent need to improve the care of SCD patients by expanding access to existing treatments, promoting the adoption of new therapies.

In Brazil, the care of patients with SCD in the public National Health Service (*Sistema Único de Saúde* - SUS) is guided by the Brazilian Clinical Practice Guidelines (CPG). These guidelines outline comprehensive disease management and regulate the use of health technologies listed in the NHS, specifying eligibility criteria, monitoring requirements, and conditions for treatment discontinuation. The NHS is a publicly funded healthcare system that provides universal access to listed health services, including prevention, diagnosis, treatment, and rehabilitation. When a health technology is listed in the NHS, it must be provided free of charge to patients. Its distribution and availability may occur at the federal, state, or municipal level, depending on the management and procurement policies established.

This work aims to present the process of updating the CPG for SCD, including the health technology assessment process and final recommendations on diagnostic and therapeutic approaches.

## Methods

The updated Brazilian CPG for the diagnosis, treatment, and monitoring of SCD patients was developed by the Ministry of Health (MoH) in conjunction with invited medical experts and methodological researchers from the Health Technology Assessment Unit at the Hospital Alemão Oswaldo Cruz. Although no clinical protocols aligned with our scope or recently updated CPG were identified to support the adoption (adolopment) process recommended by the GRADE-ADOLOPMENT methodology [22], in this update we reviewed previous SCD CPG recommendations (from 2018) [23] and considered the addition of new recommendations when pertinent to the Brazilian context, and new scientific evidence available in the literature. The entire process of updating and drafting the guidelines followed the legislative and methodological guidelines established by the MoH [24], which are aligned with the GRADE guidelines [25].

The AGREE II Reporting Checklist was used to support reporting guideline recommendations (Supplementary Table S1).

### Scope and purpose

The target population includes children and adults with suspicion or diagnosis of SCD, whether with the sickle cell trait (Hb AS), the homozygous Hb S mutation (Hb SS), or compound heterozygosity (e.g., Hb Sβ⁰, Hb Sβ⁺, Hb SC, Hb SD, Hb Sβ^thal^, according to the International Classification of Diseases-10 (ICD-10) codes D57.0, D57.1, D57.2.

### Guidelines development group

The CPG Development Group was composed of a multidisciplinary team consisting of researchers, clinical experts, and policymakers to ensure a comprehensive and evidence-based approach. The process was coordinated by the General Coordination of Clinical Protocols and Therapeutic Guidelines (CGPCDT), a division of the Brazilian MoH responsible for the development and revision of CPGs (management committee) [26].

The researchers were from the Health Technology Assessment Unit (UATs) of the Hospital Alemão Oswaldo Cruz, a recognized Center with expertise in health technology assessment (HTA) and pharmacoeconomics (drafting group). Researchers were responsible for mapping relevant technologies for reimbursement, conducting systematic literature reviews and full HTA, including cost-effectiveness and budget impact analyses.

The clinical specialists (panelist group) were selected based on their expertise in hematology and SCD care. They contributed to the definition of the scope of the guidelines, formulation of PICO (Population, Intervention, Comparator, Outcome) questions, and drafting of clinical recommendations.

The government representatives included technical advisors of the MoH and representatives from the Secretariat of Science, Technology, and Innovation. The Health Economic-Industrial Complex (SECTICS), Department of Specialized and Thematic Care (DAET), and Department of Pharmaceutical Assistance and Strategic Supplies (DAF) provided strategic advice and ensured alignment with public health priorities. The final version of the CPG was reviewed by CGPCDT to ensure consistency and alignment with MoH regulations.

### Meetings and updating process

The selection of CPG for periodic updates follows predefined criteria established by the Brazilian MoH. These include the time since the last update, availability of new evidence, listing of new health technologies in the NHS, horizon scanning studies and recommendations from the MoH technical areas [26].

The CPG development process began with a broad literature mapping and identification of international protocols. Initially, three virtual meetings were held between June and August 2022 involving the CPG Development Group.

The first preliminary meeting focused on defining the scope of the CPG and identified priority topics for revision. During this phase, researchers presented a synthesized overview of the literature mapped, along with a comparative analysis of key recommendations from international CPGs and the current version of the national CPG.

The second meeting delved deeper into clinical aspects, including the discussion of eligibility criteria and key recommendations for diagnosis, treatment, and monitoring. During this meeting, medical specialists identified specific sections of the CPG for revision or rewriting, ensuring that the document reflects current clinical standards and practices [27].

The final meeting was dedicated to prioritizing PICO questions related to the technologies listed in the NHS that required evaluation in order to be included in the CPG. These technologies, either diagnostic tools or treatments, were typically identified through the initial literature search. This step ensured that the guidelines incorporate relevant, evidence-based technologies that enhance disease management and align with national healthcare priorities.

Subsequently, two parallel processes were initiated. While clinical specialists began revising the existing text and drafting the updated CPG, researchers undertook the HTA process for the prioritized technologies to be evaluated for reimbursement. This dual approach ensured that the CPG text and the economic and clinical assessments were developed concurrently, allowing a seamless integration of evidence-based recommendations and updated technological evaluations.

### Health technology assessment

The National Committee for Health Technology Incorporation (Conitec) plays a critical role in the evaluation and recommendation of health technologies for inclusion in the NHS. Conitec is responsible for assessing the efficacy, safety, cost-effectiveness, and budgetary impact of medicines, devices, and other health technologies proposed for use within the NHS, as well as to approve the publication of the CPGs.

For this reason, researchers conducted the entire HTA process following the methodological guidelines of the MoH [28, 29, 30], including systematic reviews, cost-effectiveness and budgetary impact analyses of technologies prioritized during the third meeting. These findings were then presented and discussed in plenary meetings of Conitec, which included representatives from various governmental and technical bodies. The commission deliberated on the evidence and formulated recommendations considering the benefits and harm, values and preferences, resource use, acceptability, cost and feasibility with all these aspects being comprehensively described in the published reports [31, 32, 33]. Although the assessment of Conitec and recommendation process was not conducted by an expert panel, the evaluation of health technologies considered key domains aligned with the GRADE Evidence to Decision (EtD) framework. Therefore, the results are presented in accordance with this structure in the present article.

All aspects related to evidence retrieval, economic evaluations, and the criteria considered by Conitec during plenary discussions for recommending listed health technologies are detailed in the respective recommendation reports, which are publicly available on the website of the agency [34]. These recommendation reports comprehensively document key methodological aspects, including search methods, evidence selection criteria, strengths and limitations of the evidence and the consideration of benefits and harm. These aspects have been summarized in Supplementary Table S2.

In parallel, clinical specialists drafted the CPG based on the discussions and agreements from the preliminary meetings. If Conitec recommended a technology for inclusion in the NHS, the CPG draft was updated to reflect the technologies listed. This included specifying criteria for patient inclusion and exclusion, usage recommendations, and monitoring requirements, ensuring that the CPG remained aligned with national healthcare priorities.

### Public consultation and final recommendations

After the initial presentation of HTA in the plenary meetings of Conitec, preliminary recommendations underwent a public consultation phase to gather input from stakeholders and to capture the views and preferences of patients and the public, further ensuring that the final decision aligns with societal needs and values. During this phase, the public, including patients, caregivers, healthcare professionals, and other stakeholders, were invited to provide feedback. Contributions could be submitted via an online platform, and all the input was systematically reviewed by the drafting group and presented to Conitec [35].

Conitec was also responsible for preliminary and final recommendations of the CPG, which also went through the process of evaluation, recommendation, and public consultations. The updated CPG was subsequently subjected to a Public Consultation (No. 25/2024) held from May 24 to June 12, 2024. A total of 184 contributions were received and reviewed, influencing the final recommendations of the CPG [36].

## Recommendations

The target users of the CPG are the healthcare professionals involved in the care of SCD patients, particularly physicians and nurses working in primary and specialized care, including outpatient settings within the NHS.

**Figure 1:**
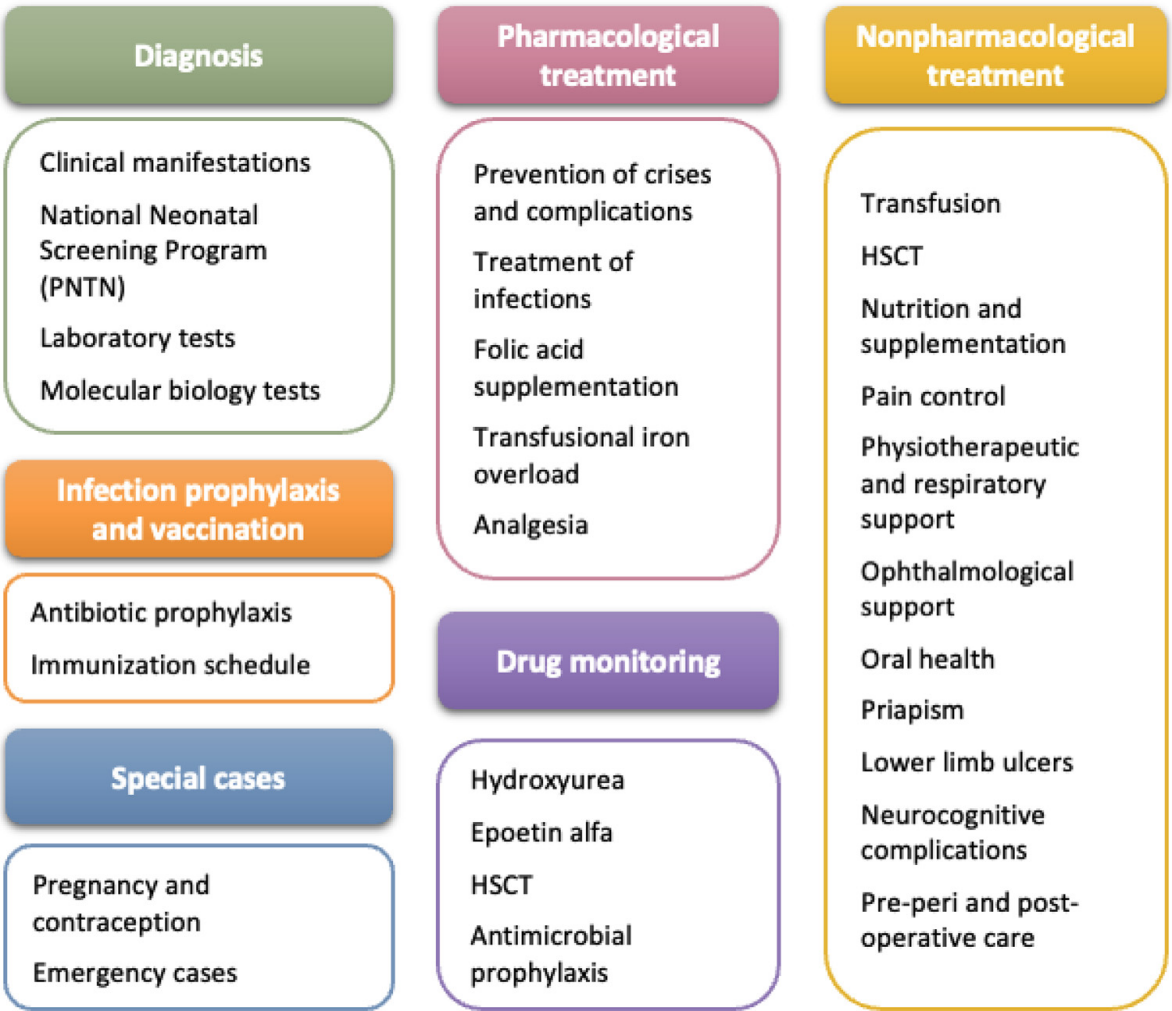
Overview of major topics for recommendations in the updated national guidelines.

SCD treatment requires coordinated actions by a multidisciplinary team for the prevention of crises, infection and complications, and for this reason, these CPG cover key aspects of screening, diagnosis, and disease identification, as well as prophylaxis and vaccination strategies. They also outline recommended actions and clinical approaches for healthcare professionals, patients, and families, addressing both pharmacological and non-pharmacological dimensions of comprehensive SCD care (Figure 1). Educating patients on self-care and family members on recognizing emergencies is of utmost importance to ensure early care, particularly for acute conditions such as fever and autosplenectomy.

Expected benefits from the treatments and monitoring outlined in these CPG include: (i) elimination or reduction of pain episodes; (ii) increased fetal hemoglobin (Hb F) production; (iii) slight increase in total Hb concentration; (iv) reduced acute chest syndrome (ACS) episodes; (v) reduced hospitalizations; (vi) reduced transfusions; (vii) regression or stabilization of organ or tissue damage; (viii) lower infection risk; and (ix) improved well-being, quality of life, and survival rates.

### Diagnosis

#### Clinical presentation

SCD presents diverse clinical manifestations that aid in diagnosis, including signs of anemia (pallor, jaundice, fatigue, tachycardia, and heart murmurs), vaso-occlusive episodes, and renal complications that may progress to chronic kidney failure, with consequences such as glomerular hyperfiltration, hyposthenuria, and the need for hemodialysis [3, 4, 5, 6]. Frequent infections, especially in young children, are the leading cause of mortality due to functional asplenia, which increases susceptibility to pathogens like *Streptococcus pneumoniae*. Severe crises, such as splenic sequestration, can result in hypovolemic shock and risk of death [37, 38, 39].

#### National neonatal screening program

SCD can be diagnosed at birth through the National Neonatal Screening Program (national policy for the newborn blood spot test) [40] or later in children and adults who did not have access to neonatal screening. It is recommended that detection and initiation of treatment occur before four months of age to ensure proper prevention of infections and other potentially fatal complications.

#### Laboratory tests

Hematological abnormalities can be observed in the complete blood count of SCD individuals, including reduced mean corpuscular volume and mean corpuscular hemoglobin concentration, as well as increased leukocyte counts and reticulocyte counts (ranging from 4-10 %). In optical microscopy of blood smears, additional features may include sickle-shaped RBCs, codocytes, hypochromia, microcytosis, polychromasia, Howell-Jolly bodies, and Heinz bodies. While these findings may assist in clinical suspicion for late diagnosis, they are not confirmatory of the disease.

Hemoglobin electrophoresis using isoelectric focusing or high-performance liquid chromatography is recommended for confirming the diagnosis, as both are accurate and usually do not require repeat testing. Conventional hemoglobin electrophoresis on agarose gel or cellulose acetate can also be used in adults. Inconclusive results from any method may be clarified with a complementary technique. For neonatal screening, repeat testing, if required, should use direct patient samples to prevent sample mix-ups.

#### Molecular biology tests

Molecular biology tests, such as polymerase chain reaction (PCR), should be incorporated into the diagnostic process to elucidate the genotype and identify polymorphisms, which are important for understanding the prognosis and severity of the disease [41].

The quantification of Hb A_2_ and Hb F is recommended, as well as familial analysis of the parents and siblings, and the identification of alpha-thalassemia variants, haplotypes associated with the Hb S gene, and other Hb variants [14].

Primary healthcare services, upon receiving the newborn blood spot test result or the hemoglobin electrophoresis test result, must initiate care for SCD individuals. The diagnostic and referral workflow are represented in Supplementary Figure S1.

### Infection prophylaxis and vaccination

Daily antibiotic prophylaxis is recommended for children with asplenia or hyposplenism until age five or at least one-year post-splenectomy. High-risk groups, including children up to 16 years, adults over 50, immunocompromised patients, and those with prior sepsis, should prioritize prophylaxis. For over five-year-old patients without high-risk features, 1-2 years of prophylaxis post-splenectomy may suffice [42,43].

Oral penicillin V is the preferred agent, with injectable penicillin G as an alternative when oral administration is not possible. For penicillin-allergic children, erythromycin is recommended. Amoxicillin and penicillin are first-line choices, while cephalosporins, fluoroquinolones, and macrolides are alternatives (Table 1).

**Table 1:** Recommended prophylactic treatment for children aged three months to five years.

Drug	Criteria	Dosage regimen
Oral penicillin V (phenoxymethylpenicillin)	Children under three years old or weighing up to 15 kg	125 mg (equivalent to 200,000 IU or 2.5 mL) every 12 h (250 mg/day)
	Children over three years old or weighing between 15 and 25 kg	250 mg (equivalent to 400,000 IU or 5 mL) every 12 h (500 mg/day)
Intramuscular benzathine penicillin G (benzylpenicillin) *	Patients weighing up to 10 kg	300,000 IU every 4 weeks
	Patients weighing between 10 and 20 kg	600,000 IU every four weeks
	Patients weighing over 20 kg	1,200,000 IU every four weeks
Oral erythromycin estolate	For penicillin allergy	20 mg/kg twice daily (40 mg/kg/day)

⁎ IM: Intramuscular; IU: international units

Vaccination should align with the MoH schedule, including 23-valent pneumococcal polysaccharide vaccine (PPSV23) and 13-valent pneumococcal conjugate vaccine (PCV13) after age two, with a booster after 3-5 years [44]. An annual influenza vaccination is recommended for all ages, and COVID-19 vaccination is advised for children and adults with SCD [44].

### Non-pharmacological treatment

#### Transfusion support

Transfusion support is essential for acute and chronic disease management, as well as during pregnancy and in perioperative periods [45,46]. Transfusion strategies include simple transfusion, which restores volume and oxygen capacity but risks volume and iron overload, and exchange transfusion, which removes sickled RBCs and replaces them with normal RBCs through erythrocytapheresis or partial manual exchange. Indications for each approach depend on the patient’s clinical condition, including age, comorbidities, transfusion history, and treatment alternatives (Table 2) [45, 46, 47]. It is recommended to avoid unnecessary transfusions to reduce risks like alloimmunization and iron overload, and monitor serum ferritin levels, especially in irregular transfusions.

**Table 2:** Indications for each type of transfusion.

Simple transfusion	Exchange transfusion
• Aplastic crisis and pancytopenia; • Acute and progressive infections with a hemoglobin drop of 1.5 g/dL from baseline or Hb below 7 g/dL; • Acute hepatic or splenic sequestration; • Pregnancy; • Acute stroke when exchange transfusion is unavailable; • Acute chest syndrome (ACS) with increasing oxygen requirement to maintain saturation above 95 %.	• Drop in baseline Hb with symptomatic anemia or acute Hb drop without reticulocytosis; • ACS, with transfusion continuing for at least six months post-episode; • Frequent and severe pain crises; • Acute stroke or cerebral infarction; • Transient ischemic attack (TIA); • Multi-organ failure; • Priapism unresponsive to urological procedures; • Increased velocity on transcranial Doppler; • Preparation for major surgeries or procedures involving vital organs.

Phenotyping for ABO, Rh, and Kell antigens is required at diagnosis, with transfused RBCs matched to prevent sensitization. If antibodies develop, extended phenotyping is needed. Immunohematology tests are available through public blood centers, and confirmatory testing is advised after six months of age and before the first transfusion [47,48].

Leukoreduced, phenotyped, and sickle cell-free RBCs are recommended for all transfusions. In rare cases, RBCs with sickle cell trait may be used for unique antigen profiles. Exchange transfusions are preferred over simple transfusions when feasible to minimize iron overload and alloimmunization. For patients eligible for hematopoietic stem cell transplantation (HSCT), irradiated blood components should be used, following MoH guidelines [49].

#### Hematopoietic stem cell transplantation

Myeloablative allogeneic HSCT from an HLA-matched sibling or haploidentical donor, or using cord blood, peripheral blood, or bone marrow is recommended for treating SCD in patients with the Hb SS or Hb Sβ^0^ genotypes experiencing severe vaso-occlusive complications despite HU treatment. HLA testing should begin at age five or upon the onset of severe complications to identify potential candidates. Transplantation age criteria are not explicitly defined, and the choice of stem cell source should balance donor and recipient risks [50,51].

#### Strategies for pain management

Families and individuals with SCD should prioritize hydration and avoid vaso-occlusive crisis triggers, such as temperature changes, infections, menstruation, pregnancy, and stress [52]. Non-pharmacological pain management includes psychosocial support, cognitive-behavioral therapy, relaxation techniques, and addressing contributing factors like stress, insomnia, and malnutrition. Individualized care plans are crucial for managing chronic and acute pain [53,54].

Physical activity is beneficial but requires medical guidance. Light exercises with gradual progression, stretching, and breaks every 20 min are recommended to avoid fatigue and pain. Activities like walking, swimming, and cycling at 50-90 % maximum heart rate for 20 min are ideal. Hydration, proper nutrition, and avoiding prolonged or intense workouts are essential [52,55,56].

#### Nutrition and supplementation

A balanced diet rich in vitamins A, C, and E, as well as minerals such as zinc and copper are recommended. Bone health should be evaluated regularly, including assessments of calcium intake and annual or semiannual vitamin D levels [57]. Bone density measurements should begin at age 12, with vitamin D assessments repeated annually and bone density testing every three years. Supplementation with vitamin D and calcium can support the bone metabolism, helping to prevent growth and maturation delays in children and adolescents with SCD [58,59].

#### Physiotherapeutic and respiratory support

Aerobic kinesiotherapy, analgesic electrotherapy, and electroacupuncture may benefit SCD patients. Continuous incentive spirometry is recommended during hospital stays or when ACS develops [60]. For patients with increasing oxygen needs or reduced respiratory effort, non-invasive ventilation techniques like Continuous/Bilevel Positive Airway Pressure (CPAP/BiPAP) can be beneficial [61,62].

#### Oral health

Periodic dental evaluations are essential for SCD patients. Preventive care, monitoring, and treatment of maxillofacial alterations, such as malocclusion, help reduce disease-associated complications [63,64].

#### Ophthalmological support

Patients with the Hb SS, Hb Sβ^0^, and Hb SC genotypes are most significantly affected, particularly within the 20-39 year age group; therefore, annual ophthalmological monitoring should begin at age ten and continue throughout adulthood [65].

#### Priapism

Prophylactic measures for priapism include increased fluid intake and frequent bladder emptying. For episodes under two hours, hydration, bathing, walking, warm compresses, and analgesia are recommended. Priapism lasting more than 2-4 h is a medical emergency requiring immediate evaluation, hydration, analgesia, and a urological consultation [66,67].

#### Leg ulcers

Wound care involves keeping the skin hydrated and using appropriate footwear. Ulcers should be cleaned with 4 % sodium chloride solution using specific techniques based on wound type (e.g., jet irrigation for clean wounds, mechanical cleaning with dry gauze for infected wounds or wounds with debris). Dexamethasone cream and mineral oil are recommended for perilesional skin, with mineral oil use continuing post-healing. The tetanus vaccination should be updated [68].

#### Neurocognitive complications

Stroke prevention includes annual transcranial Doppler ultrasound for patients aged 2-16 years to assess risk [69]. Increased cerebral artery velocity indicates a higher stroke risk, requiring chronic transfusion therapy to lower Hb S levels. Selective screening with cerebral magnetic resonance angiography may also be needed.

Silent strokes, defined as lesions visible on at least two planes by T2-weighted magnetic resonance imaging (MRI), measuring at least 3 mm in the largest linear dimension [70], without associated neurological findings, can be detected early by cognitive tests conducted by qualified psychology professionals, and should be included in care for children and adolescents with SCD, enabling measures to mitigate these consequences [71, 72, 73, 74].

#### Surgical procedures in sickle cell disease

SCD patients require special care before, during, and after surgical procedures [75], as presented in Table 3.

**Table 3:** Recommended care for patients with sickle cell disease undergoing surgical procedures.

Preoperative care	Perioperative care	Postoperative care
• Clinical evaluation; • Complete blood count and coagulation tests; • Glucose, urea, creatinine, liver function tests, and urinalysis; • Electrocardiogram (ECG) for surgical cardiac risk assessment; • Chest X-ray; • Hb A and Hb S quantification; • Pulse oximetry and respiratory physiotherapy evaluation; • Transfusion preparation and immuno-hematological testing with red blood cell phenotyping; • Full hydration for 12 hours preoperatively	• Maintaining a moderate temperature in the operating room; • 50 % oxygenation combined with the anesthetic agent; • Clinical monitoring (ECG, blood pressure, pulse, temperature, urinary output); • Laboratory monitoring (serum electrolytes, inspired O^2^ concentration, pulse oximetry, or arterial blood gas analysis).	• Immediate postoperative oxygenation; • Pulse oximetry monitoring; • Parenteral hydration; • Respiratory physiotherapy.

### Pharmacological treatment

Four research questions for listing technologies were prioritized for the updated CPG, for which the working group developed recommendation reports (Table 4). As a result of the HTA process and Conitec deliberation, epoetin alfa [33] and presentation of HU in 100 mg tablets [32] were listed in the NHS. Additionally, the criteria for the use of HU were expanded [31], allowing broader access to treatment for SCD patients (Supplementary Table S2). Crizanlizumab was also prioritized for assessment; however, its evaluation was suspended, and the assessment report did not proceed to the plenary deliberation of Conitec following the cancellation of its marketing authorization by National Health Surveillance Agency (Anvisa) on October 30, 2023. The eligibility criteria for using listed treatments were established in accordance with the listing recommendations of Conitec, and the original text was revised to prevent access barriers. (Supplementary Table S3).

**Table 4:** Summary of health technology prioritized and assessed for coverage decisions.

Technology and research question	Clinical evidence*	Economic assessments	Coverage decision
Epoetin alfa for reduced kidney function	Eight observational studies analyzed before/after clinical and hematological outcomes. Epoetin alfa use significantly increased: • Hb levels (4-32.8 % improvement, p<0.05) (certainty of evidence: **VERY LOW**) • Hb F levels (5.2-17.1 % increase, p<0.05), and reduced transfusion requirements (quantitative data not reported) (certainty of evidence: **VERY LOW**) No increase in vaso-occlusive crises (VOC) or venous thromboembolism (VTE) was observed, suggesting safety (quantitative data not reported) (certainty of evidence: **VERY LOW**) For a renal impairment subgroup (*n* = 4), Hb concentration improved by 29 % (certainty of evidence: **VERY LOW**)	A CEA using a one-year decision tree model evaluated its impact on reducing transfusion needs. Epoetin alfa showed modest clinical benefit (incremental 0.033 QALY) and cost savings of BRL 11,564. Sensitivity analyses confirmed epoetin alpha as a dominant option. The eligible population for the BIA was estimated at 5,274 patients annually. With a 10-50 % market share, direct acquisition costs was BRL 806,129 in year one to BRL 4.8 million in year five. Over five years, a cumulative savings of BRL 96.5 million, due to reduced transfusion-related costs, including iron chelation therapy.	After public consultation, Conitec members unanimously recommended incorporating epoetin alfa into the NHS for treating SCD patients with renal impairment and worsening hemoglobin levels, following the Ministry of Health’s clinical protocol. The decision was formalized under Resolution No. 871/2024.
Hydroxyurea to nine-month-old children	A randomized clinical trial (BABY HUG - NCT00006400) and three non-comparative observational studies were included as clinical evidence. Hydroxyurea showed a significant impact on the following efficacy outcomes compared to placebo: • Hb levels (difference of 0.9; 95 % CI: 0.5-1.3; p<0.001) (certainty of evidence: **MODERATE**) • Hb F levels (difference of 6.7 %; 95 % CI: 4.8-8.7 %; p<0.001) (certainty of evidence: **MODERATE**) • Pain (HR 0.59; 95 % CI: 0.42-0.83; *p* = 0.002) (certainty of evidence: **MODERATE**) • Acute chest syndrome (HR 0.36; 95 % CI: 0.15-0.87; *p* = 0.02) (certainty of evidence: **MODERATE**) • Transfusion requirements (HR: 0.55; 95 % CI: 0.32-0.96; *p* = 0.03) (certainty of evidence: **MODERATE**) No significant difference was found for hospitalizations (HR: 0.73; 95 % CI: 0.53-1.00; *p* = 0.05) (certainty of evidence: **MODERATE**)	In the CEA, hydroxyurea was cost-effective when considering QALY and may be cost-effective (ICER – BRL 12,258 per QALY gained). In BIA, it was estimated that the 5-year cumulative impact for the adoption of hydroxyurea for patients aged 9-24 months could range from BRL 105,556 to BRL 484,805.	After a public consultation, the Conitec members unanimously recommended incorporating hydroxyurea in the treatment of patients with sickle cell SCD (Hb SS, Hb Sβ0, severe Hb Sβ+, and Hb SD Punjab), aged 9-24 months, without symptoms or complications, considering the clinical benefits and favorable budget impact.
Hydroxyurea 100 mg tablet and 1000 mg tablet	It was considered that the new formulations (100 mg and 1000 mg) are bioequivalent or similar to the 500 mg formulation, and, for this reason, only economic only viability assessment was made. Certainty of the evidence was not assessed since the studies were based on bioequivalence analyses.	In the BIA, two populations were considered: individuals >9 months (17,400 per year) and 9 months to 12 years (5,000 per year). With a 10-50 % market share, the 5-year impact would be approximately BRL 396.7 million and BRL 43 million, respectively.	After public consultation, the members of Conitec unanimously recommended the incorporation of 100 mg hydroxyurea and not to incorporate 1000 mg hydroxyurea for the treatment of patients with SCD aged at least 9 months. The Committee maintained this decision, considering that the potential clinical benefits of using the 1000 mg form would not justify its costs.

BIA: budget impact analysis; BRL: Brazilian Reais; CEA: cost-effectiveness analysis Hb: Hemoglobin; Hb F: Fetal hemoglobin; ICER: incremental cost-effectiveness ratio; 95 % CI: 95 % confidence interval; HR: Hazard ratio.

⁎ Further aspects of the GRADE Evidence-to-Decision (EtD) framework, considered in the recommendations reports and discussed during the meetings of Conitec are presented in the Supplementary Material.

### Prevention of crises and complications

HU is the cornerstone of SCD management and the most effective pharmacological therapy, requiring regular monitoring due to a risk of hematologic toxicity albeit low [76,77]. While HU has shown in-vitro carcinogenic and teratogenic potential, this has not been confirmed in vivo [78]. Treatment should continue indefinitely if clinical and laboratory responses are achieved [79]. For about 25 % of patients with an unsatisfactory response, discontinuation may be considered after two years. Treatment adherence should be reassessed to optimize outcomes, with healthcare teams emphasizing the benefits of HU to the patient.

The recommended starting dose of HU is 15 mg/kg/day, taken orally as a single daily dose. The dose can be increased by 5 mg/kg/day every four weeks, up to a maximum of 35 mg/kg/day or until hematological toxicity or serious adverse effects occur. For children weighing up to 25 kg, the 100 mg coated tablet is advised.

Recombinant human epoetin alfa is recommended for Hb SS and Hb Sβ^0^ patients on HU who experience declining hemoglobin levels or frequent transfusion needs. The suggested dose is 12,000 IU per week, divided into three administrations of 4,000 IU each, delivered via subcutaneous or intravenous injection [80,81].

#### Treatment of Infections

Prompt recognition and treatment of infections are critical due to the high risk of septic shock. Fever is an emergency, especially in under three-year-old children with temperatures above 38.2°C. Signs of infection require immediate transport to an emergency facility and a Reference Center notification. Diagnostic tests include blood count, reticulocytes, urinalysis, c-reactive protein, cultures, chest X-ray, and lumbar puncture if suspicion of central nervous system infection. Empirical antibiotics for encapsulated organisms should start immediately for undiagnosed febrile cases. Antibiotic selection varies by infection type:

• ACS: Add macrolides (e.g., clarithromycin, azithromycin).
• Meningitis: Use CNS-penetrating antibiotics (e.g., ceftriaxone).
• *Mycoplasma pneumoniae*: Add erythromycin, clarithromycin, or azithromycin.
• Osteomyelitis: Provide 4-6 weeks of antibiotics covering *S. aureus* and *Salmonella spp*. along with hydration and analgesia.

SCD patients with COVID-19 require close monitoring, hospitalization at the first complication sign, and careful management even with mild symptoms [82].

#### Folic acid supplementation

Folic acid is recommended for low serum folate levels or specific conditions such as pregnancy, with a dosage of 1 mg/day. Serum folic acid levels should be maintained below 17 ng/mL. If therapeutic monitoring is unavailable, dietary guidance is advised alongside supplementation. For children up to 1 year or 10 kg, a half tablet (2.5 mg) three times per week is recommended. For patients above this age or weight, one tablet (5 mg) three times per week is advised.

#### Transfusional iron overload

Treatment should follow the current iron overload CPG of the MoH [83].

#### Analgesia

Pain management during vaso-occlusive crises requires fixed-dose analgesics based on intensity, guided by pain scales which can direct treatment, monitor responses, and predict hospitalization duration [84,85]. Treatment must be individualized to avoid opioid toxicity when alternative therapies are more appropriate, following the current National CPG for Chronic Pain [86]. Chronic pain from avascular necrosis, compression fractures, or arthropathies may require surgical interventions. Menstrual pain may benefit from hormonal therapies or NSAIDs, while priapism requires adequate analgesia pending a urological evaluation [87].

#### Iron supplementation

Iron supplementation is not routinely recommended and should only address specific deficiencies identified by ferritin, iron, and transferrin levels, with treatment limited to correcting the deficiency [88].

### Special cases

#### Pregnancy and contraception

Pregnant women with SCD face higher risks of placental dysfunction, infections, preterm birth, and perinatal mortality. Untreated bacteriuria can cause intrauterine growth restriction (IUGR) and preterm labor, while anemia may worsen due to increased blood demand, hemodilution, or deficiencies. Women should be informed about risks such as IUGR, preeclampsia, eclampsia, infections, thromboembolism, gestational diabetes, and preterm delivery. Miscarriages are more common, especially in Hb SS patients, and delayed puberty may postpone pregnancy.

High-risk obstetric care, including ultrasound, fetal biophysical profile, umbilical Doppler, and cesarean delivery, when necessary, is essential to reduce perinatal mortality. Regular prenatal visits, hydration, and timely management of fever or pain crises are critical. Pregnant women must interrupt unsafe medications such as iron chelators and discontinue HU three months before conception. Folic acid (5 mg/day) is recommended during pregnancy.

Pain crises, more frequent late in pregnancy, are treated similarly to non-pregnant patients. NSAIDs may be used between 18-30 weeks but should be avoided after 30 weeks to prevent ductal closure. Routine monthly transfusions are reserved for severe cases, while intermittent transfusions may address significant anemia (<7 g/dL).

Initial care includes clinical and obstetric history, paternal Hb electrophoresis, and comprehensive lab tests. Monitoring at each trimester and updating maternal immunizations are recommended. Maternal assessments should cover nutrition, hydration, blood pressure, weight gain, and cervical evaluation.

Progestogen-only contraceptives, like medroxyprogesterone acetate, are preferred due to lower thrombosis risk. Copper intrauterine devices are an option but require monitoring for increased menstrual flow. Bone density should be evaluated before starting progestogen-based contraceptives and reassessed every two years.

#### Emergency cases

ACS is identified by new pulmonary infiltrates on chest X-ray and symptoms like chest pain, cough, fever, hypoxemia, or respiratory distress. Urgent hospitalization is required, with intensive care unit transfer if oxygen saturation falls below 93 %. Management includes oxygen therapy to maintain saturation ≥93 %, monitoring blood counts, reticulocytes, blood cultures, and chest X-rays. Bronchodilators and respiratory physiotherapy may be used. Fluid management should avoid overhydration to prevent pulmonary edema. Simple RBC transfusion is indicated if Hb drops by ≥1.5 g/dL below baseline. For severe or rapidly progressing cases, exchange transfusion or urgent simple transfusion is recommended. Post-recovery, exchange transfusion should continue for at least six months [89].

Splenic Sequestration progresses rapidly and requires immediate hospitalization and notification of a reference center. Families should monitor spleen size to detect recurrence, which occurs in up to 50 % of cases [90]. Treatment involves IV hydration (10-15 mL/kg plasma expanders or 40-100 mL/kg saline over two hours), bed rest, oxygen therapy, and leg elevation. RBC transfusion (10 mL/kg) may be needed to achieve Hb levels of 6-7 g/dL, with careful volume monitoring post-crisis to prevent hyperviscosity. Splenectomy is often performed after the first acute event in over five-year-old children to prevent recurrence.

Hyper-Hemolysis Syndrome (HHS) is a severe hemolysis exacerbation following transfusion, marked by a rapid Hb drop below pre-transfusion levels. Immediate treatment includes hydration and immunosuppressive therapy with glucocorticoids (1-4 mg/kg/day prednisone or equivalent methylprednisolone), IV immunoglobulin (0.4-1 g/kg/day for 3-5 days), or both. Therapy should be individualized, with prompt intervention essential to prevent fatal outcomes [91,92].

### Monitoring

#### Hydroxyurea

Monitoring HU in SCD aims to ensure clinical effectiveness and safety. Initial tests include Hb electrophoresis, Hb F levels, complete blood count (CBC), liver and kidney function tests, and pregnancy testing. During treatment, Hb F, CBC, and liver and kidney function should be monitored periodically to guide dose adjustments (Supplementary Table S4).

If toxicity occurs, HU should be discontinued until recovery and resumed at a dose 5 mg/kg/day lower than the previous dose. Recurrent toxicity at the same dose defines the maximum tolerated dose. HU dose must be adjusted for renal impairment based on creatinine clearance (if 10-50 mL/min, administer 50 % of the dose; if less than 10 mL/min, administer 20 % of the dose) [93]. Myelosuppression is the primary dose-limiting toxicity. Toxicity levels and frequency of recommended tests are described in Supplementary Table S5 [94, 95, 96, 97]. Severe mucocutaneous reactions require discontinuation, while long-term use does not affect growth or development in children with SCD.

Lactating patients should decide between discontinuing breastfeeding or treatment, considering the benefits for the mother. However, pharmacokinetic studies have reported low HU transfer to breast milk, suggesting its use may be acceptable if breastfeeding occurs at least three hours after the daily dose [98]. Therefore, the risks and benefits should be carefully assessed in these cases. Women of childbearing age should use contraception. HIV or hepatitis patients require close monitoring for potential complications.

#### Epoetin alfa

Hemoglobin levels should be monitored weekly until stabilization, then monthly for six months, and every three months thereafter. Epoetin alfa should be discontinued if no reduction in transfusion needs or failure to achieve target Hb levels (≥8.5 g/dL or an increase ≥1.5 g/dL from baseline) occurs within six months [33]. Otherwise, treatment may continue indefinitely to reduce transfusion dependency.

#### Hematopoietic stem cell transplantation

HU should be discontinued four weeks before starting conditioning and reintroduced only in cases of engraftment failure.

#### Antimicrobial prophylaxis

Prophylactic phenoxymethylpenicillin (penicillin V) should be continued from diagnosis until age 5. Asplenia is not a contraindication for vaccinations, including live vaccines. No evidence suggests vaccine-related complications in SCD patients on HU, and the benefits of reducing infections outweigh potential risks [99].

## Transition process

Adolescence is a period of considerable vulnerability, as patients face challenges inherent to their age, considering social, physical, and psychological development. For people with SCD, this stage of life is often marked by a shift in disease behavior, with increased severity observed in some individuals. This progression is largely attributed to the underlying biology and pathophysiology of SCD, which tends to worsen due to the cumulative burden of organ damage over time. The absence of a structured transition process can result in disruptions in care, with serious consequences such as increased use of emergency services, deterioration of organ function, and early death. Therefore, preparation, transfer, and integration are essential in the transition process, which should occur in an organized and planned manner [100, 101].

A planned transition is considered a quality standard in services for patients with chronic conditions. It involves not just the transfer of care - understood as the act of moving the patient from one institution or care team to another - but a broader, dynamic, and complex process. This includes evaluating the acquisition of self-management skills and implementing educational strategies and appropriate healthcare resources [101].

The main goals of a transition clinic are: (1) to improve the capacity of adolescents and young adults, with or without special healthcare needs, to manage their own care and use health services effectively; and (2) to ensure an organized process within pediatric and adult care teams for transition preparation, transfer, and integration into adult-centered healthcare. The stages of this process, clearly outlined in the updated CPG (Supplementary Figure S2), should guide services aiming to address this important gap.

## Conclusion

The updated national Guidelines for SCD is publicly available on the MoH website and has been officially published through a Joint Ordinance (SAES/SECTICS No. 16, of November 1, 2024), ensuring accessibility of healthcare professionals, patients and all the population [102]. In addition to these dissemination efforts, the present article aims to contribute to the international discussion on SCD management, describing the Brazilian approach for evidence-based guideline development.

Regarding evaluation of implementation, no formal assessment of facilitators and barriers was planned within this update. However, it is important to emphasize that the guidelines should not be regarded as a standalone document. Given the decentralized structure of the NHS, states and municipalities have the autonomy to develop complementary tools and local protocols, aligning with the national guidelines while addressing regional healthcare needs and operational constraints. Future studies assessing the real-world application of these recommendations could provide valuable insights to optimize implementation strategies and improve patient outcomes.

## Funding source

This guideline updating process was funded through a partnership with the Brazilian Ministry of Health via the Unified Health System (SUS) Institutional Development Support Program (PROADI-SUS). This manuscript was prepared by the Health Technology Assessment Unit (UATS) of the Hospital Alemão Oswaldo Cruz (HAOC).

## Contribution author

1) Conception and design of the study: LPG, LAO, RCL, HAOJ
2) Acquisition of data, or analysis and interpretation of data: LPG, MMF, LAO, CL, KNTM, SS, RCL, MRKR, DFG
3) Drafting the article: LPG, MMF, RCL, LAO
4) Revising it critically for important intellectual content: RCL, LAO, HAOJ, MCLSM
5) Final approval of the version to be submitted: LPG, MMF, RCL, LAO, HAOJ, CL, KNTM, SS, MRKR, DFG, MCLSM

## Declaration of generative ai and ai-assisted technologies in the writing process

During the preparation of this work the authors used ChatGPT in order to improve language and readability. After using this tool/service, all authors reviewed and edited the content as needed and take full responsibility for the content of the publication.

## Conflicts of interest

The authors declare that they have no conflicts of interest to disclose.

## Appendix Supplementary materials

Image, application 1

## Footnotes

Supplementary material associated with this article can be found in the online version at doi:10.1016/j.htct.2025.103964.
