# Supplementary material for: Updating the Brazilian clinical practice guidelines for sickle cell disease: Recommendations and development process
Source: Hematol Transfus Cell Ther. 2025 Aug 23;47(4):103964. doi: 10.1016/j.htct.2025.103964 (PMC12570198; doi:10.1016/j.htct.2025.103964)

**SUPPLEMENTARY MATERIAL**

**UPDATING THE BRAZILIAN CLINICAL PRACTICE GUIDELINE FOR SICKLE CELL DISEASE: RECOMMENDATIONS AND DEVELOPMENT PROCESS**

**Table S1:** AGREE Reporting Checklist 2016

| **Checklist item and description** | **Reporting criteria** | **Page** | **Comments** |
| --- | --- | --- | --- |
| DOMAIN 1: SCOPE AND PURPOSE | | | |
| 1. OBJECTIVES  Report the overall objective(s) of the guideline. The expected health benefits from the guideline are to be specific to the clinical problem or health topic. | ☒ Health intent(s) (i.e., prevention, screening, diagnosis, treatment, etc.)  ☒ Expected benefit(s) or outcome(s)  ☒ Target(s) (e.g., patient population, society) | Pages 7-8 and Figure 1 |  |
| 2. QUESTIONS  Report the health question(s) covered by the guideline, particularly for the key recommendations. | ☒ Target population  ☒ Intervention(s) or exposure(s)  ☒ Comparisons (if appropriate)  ☒ Outcome(s)  ☒ Health care setting or context | Table 4 and Table S2 | Research questions for HTA for listing health technologies. |
| 3. POPULATION  Describe the population (i.e., patients, public, etc.) to whom the guideline is meant to apply. | ☒ Target population, sex and age  ☒ Clinical condition (if relevant)  ☒ Severity/stage of disease (if relevant)  ☒ Comorbidities (if relevant)  ☒ Excluded populations (if relevant) | Page 4 | National Guideline includes SCD people of any age, sex, or severity of disease. However, eligibility criteria for listed technologies are presented in Table S3 |
| DOMAIN 2: STAKEHOLDER INVOLVEMENT | | | |
| 4. GROUP MEMBERSHIP  Report all individuals who were involved in the development process. This may include members of the steering group, the research team involved in selecting and reviewing/rating the evidence and individuals involved in formulating the final recommendations. | ☐ Name of participant  ☒ Discipline/content expertise (e.g., neurosurgeon, methodologist)  ☒ Institution (e.g., St. Peter’s hospital)  ☐ Geographical location (e.g., Seattle, WA)  ☒ A description of the member’s role in the guideline development group | Page 5 |  |
| 5. TARGET POPULATION  PREFERENCES AND VIEWS  Report how the views and preferences of  the target population were  sought/considered and what the resulting outcomes were. | ☒ Statement of type of strategy used to capture patients’/publics’ views and preferences (e.g., participation in the guideline development group, literature review of values and preferences)  ☒ Methods by which preferences and views were sought (e.g., evidence from literature, surveys, focus groups)  ☒ Outcomes/information gathered on patient/public  information  ☒ How the information gathered was used to inform the guideline development process and/or formation of the recommendations | Pages 6-7 | Social participation (at meeting of CONITEC) and public consultation are the processes used to assess the preferences and perspectives of the target population, both regarding the clinical guideline and the Recommendation Reports for the evaluated technologies. Further details on the process can be found in the relevant legislation (1). |
| 6. TARGET USERS  Report the target (or intended) users of the guideline. | ☒ The intended guideline audience (e.g. specialists, family physicians, patients, clinical or institutional leaders/administrators)  ☒ How the guideline may be used by its target audience (e.g., to inform clinical decisions, to inform policy, to inform standards of care) | Pages 7-8 |  |
| DOMAIN 3: RIGOUR OF DEVELOPMENT | | | |
| 7. SEARCH METHODS  Report details of the strategy used to search for evidence. | ☒ Named electronic database(s) or evidence source(s) where the search was performed (e.g., MEDLINE, EMBASE, PsychINFO, CINAHL)  ☒ Time periods searched (e.g., January 1, 2004 to March 31, 2008)  ☒ Search terms used (e.g., text words, indexing terms, subheadings  ☒ Full search strategy included (e.g., possibly located in appendix) | Pages 6-7 | Detailed information related to evidence retrieval, economic evaluations, and the GRADE EtD criteria considered by CONITEC during plenary discussions for recommending listing health technologies are detailed in the respective Recommendation Reports (RR), which are publicly available on the agency's website (2–4) |
| 8. EVIDENCE SELECTION CRITERIA  Report the criteria used to select (i.e., include and exclude) the evidence. Provide rationale, where appropriate. | ☒ Target population (patient, public, etc.) characteristics  ☒ Study design  ☒ Comparisons (if relevant)  ☒ Outcomes  ☒ Language (if relevant)  ☒ Context (if relevant) | Pages 6-7 and Table S2. |  |
| 9. STRENGTHS & LIMITATIONS OF THE EVIDENCE  Describe the strengths and limitations of  the evidence. Consider from the  perspective of the individual studies and  the body of evidence aggregated across all the studies. Tools exist that can facilitate the reporting of this concept. | ☒ Study design(s) included in body of evidence  ☒ Study methodology limitations (sampling, blinding, allocation concealment, analytical methods)  ☒ Appropriateness/relevance of primary and secondary outcomes considered  ☒ Consistency of results across studies  ☒ Direction of results across studies  ☒ Magnitude of benefit versus magnitude of harm  ☐ Applicability to practice context | Pages 6-7 |  |
| 10. FORMULATION OF  RECOMMENDATIONS  Describe the methods used to formulate  the recommendations and how final  decisions were reached. Specify any areas of disagreement and the methods used to resolve them. | ☐ Recommendation development process (e.g., steps used in modified Delphi technique, voting procedures that were considered)  ☐ Outcomes of the recommendation development process (e.g., extent to which consensus was reached using modified Delphi technique, outcome of voting procedures)  ☐ How the process influenced the recommendations (e.g., results of Delphi technique influence final recommendation, alignment with recommendations and the final vote) | Pages 6-7 | Detailing of discussions on the Recommendation Reports (RR), which are publicly available on the website of CONITEC (2–4). For procedures of CONITEC, refer to the relevant legislation (1). |
| 11. CONSIDERATION OF BENEFITS AND HARMS  Report the health benefits, side effects, and risks that were considered when formulating the recommendations. | ☒ Supporting data and report of benefits  ☒ Supporting data and report of harms/side effects/risks  ☒ Reporting of the balance/trade-off between benefits and harms/side effects/risks  ☒ Recommendations reflect considerations of both benefits and harms/side effects/risks | Pages 6-7 and Table S2 |  |
| 12. LINK BETWEEN  RECOMMENDATIONS AND EVIDENCE  Describe the explicit link between the  recommendations and the evidence on  which they are based. | ☒ How the guideline development group linked and used the evidence to inform recommendations  ☐ Link between each recommendation and key evidence (text description and/or reference list)  ☐ Link between recommendations and evidence summaries and/or evidence tables in the results section of the guideline | Pages 5-7 |  |
| 13. EXTERNAL REVIEW  Report the methodology used to conduct the external review. | ☒ Purpose and intent of the external review (e.g., to improve quality, gather feedback on draft recommendations, assess applicability and feasibility, disseminate evidence)  ☒ Methods taken to undertake the external review (e.g., rating scale, open-ended questions)  ☒ Description of the external reviewers (e.g., number, type of reviewers, affiliations)  ☒ Outcomes/information gathered from the external review (e.g., summary of key findings)  ☐ How the information gathered was used to inform the guideline development process and/or formation of the recommendations (e.g., guideline panel considered results of review in forming final recommendations) | Pages 6-7 | External review was carried out during public consultations. The contributions received during the public consultation were presented and discussed during the meeting with CONITEC, and are available on the agency’s website. |
| 14. UPDATING PROCEDURE  Describe the procedure for updating the guideline. | ☒ A statement that the guideline will be updated  ☒ Explicit time interval or explicit criteria to guide decisions about when an update will occur  ☒ Methodology for the updating procedure | Page 5 |  |
| DOMAIN 4: CLARITY OF PRESENTATION | | | |
| 15. SPECIFIC AND UNAMBIGUOUS  RECOMMENDATIONS  Describe which options are appropriate in  which situations and in which population  groups, as informed by the body of evidence. | ☒ A statement of the recommended action  ☒ Intent or purpose of the recommended action (e.g., to improve quality of life, to decrease side effects)  ☒ Relevant population (e.g., patients, public)  ☒ Caveats or qualifying statements, if relevant (e.g., patients or conditions for whom the recommendations would not apply)  ☒ If there is uncertainty about the best care option(s), the uncertainty should be stated in the guideline | Pages 8-17 |  |
| 16. MANAGEMENT OPTIONS  Describe the different options for managing the condition or health issue. | ☒ Description of management options  ☒ Population or clinical situation most appropriate to each option | Pages 9-16 and Figure 1 |  |
| 17. IDENTIFIABLE KEY  RECOMMENDATIONS  Present the key recommendations so that  they are easy to identify. | ☐ Recommendations in a summarized box, typed in bold, underlined, or presented as flow charts or algorithms  ☒ Specific recommendations grouped together in one section | Pages 8-17 |  |
| DOMAIN 5: APPLICABILITY | | | |
| 18. FACILITATORS AND BARRIERS TO APPLICATION  Describe the facilitators and barriers to the guideline’s application. | ☐ Types of facilitators and barriers that were considered  ☒ Methods by which information regarding the facilitators and barriers to implementing recommendations were sought (e.g., feedback from key stakeholders, pilot testing of guidelines before widespread implementation)  ☒ Information/description of the types of facilitators and barriers that emerged from the inquiry (e.g., practitioners have the skills to deliver the recommended care, sufficient equipment is not available to ensure all eligible members of the population receive mammography)  ☒ How the information influenced the guideline development process and/or formation of the recommendations | Pages 6-7 and Table S2 | Facilitators and barriers for implementation was specifically assessed related to the technologies listed (2–4). However, considering the organizational structure of SUS, all recommendations were made in alignment with the capacity for implementation and delivery of healthcare services across the municipal, state, and federal levels. |
| 19. IMPLEMENTATION ADVICE/TOOLS  Provide advice and/or tools on how the recommendations can be applied in practice. | ☒ Additional materials to support the implementation of the guideline in practice. For example:  o Guideline summary documents  o Links to check lists, algorithms  o Links to how-to manuals  o Solutions linked to barrier analysis (see Item 18)  o Tools to capitalize on guideline facilitators (see Item 18)  o Outcome of pilot test and lessons learned | Figure S1 |  |
| 20. RESOURCE IMPLICATIONS  Describe any potential resource implications of applying the recommendations. | ☒ Types of cost information that were considered (e.g., economic evaluations, drug acquisition costs)  ☒ Methods by which the cost information was sought (e.g., a health economist was part of the guideline development panel, use of health technology assessments for specific drugs, etc.)  ☒ Information/description of the cost information that emerged from the inquiry (e.g., specific drug acquisition costs per treatment course)  ☒ How the information gathered was used to inform the guideline development process and/or formation of the recommendations | Pages 6-7 and Table S2 | Resource implications were assessed only for newly listed technologies, as the recommendations primarily guide the care already provided within the healthcare system. Detailed information can be access at the Recommendation Reports (RR), which are publicly available on the agency's website (2–4). |
| 21. MONITORING/ AUDITING CRITERIA  Provide monitoring and/or auditing criteria to measure the application of guideline recommendations. | ☐ Criteria to assess guideline implementation or adherence to recommendations  ☐ Criteria for assessing impact of implementing the recommendations  ☐ Advice on the frequency and interval of measurement  ☐ Operational definitions of how the criteria should be measured | Pages 17-18 |  |
| DOMAIN 6: EDITORIAL INDEPENDENCE | | | |
| 22. FUNDING BODY  Report the funding body’s influence on the content of the guideline. | ☒ The name of the funding body or source of funding (or explicit statement of no funding)  ☐ A statement that the funding body did not influence the content of the guideline | Page 18 |  |
| 23. COMPETING INTERESTS  Provide an explicit statement that all group members have declared whether they have any competing interests. | ☐ Types of competing interests considered  ☐ Methods by which potential competing interests were sought  ☒ A description of the competing interests  ☐ How the competing interests influenced the guideline process and development of recommendations | Page 18-19 |  |

Adapted from Brouwers MC, Kerkvliet K, Spithoff K, on behalf of the AGREE Next Steps Consortium. The AGREE Reporting Checklist: a tool to improve reporting of clinical practice guidelines. BMJ 2016;352:i1152. doi: 10.1136/bmj.i1152.

**References**

1. Brasil. Ministério da Saúde. Portaria no 2.009, de 13 de setembro de 2012. Aprova o Regimento Interno da Comissão Nacional de Incorporação de Tecnologias no Sistema Único de Saúde (CONITEC). [Internet]. 2012 [cited 2025 Feb 25]. Available from: https://bvsms.saude.gov.br/bvs/saudelegis/gm/2012/prt2009_13_09_2012.html

2. Brasil. Ministério da Saúde. Secretaria de Ciência, Tecnologia e Inovação e do Complexo Econômico-Industrial da Saúde - SECTICS. Relatório de Recomendação No 873 - Hidroxiureia para o tratamento de pacientes com doença falciforme (SS, Sbeta0 e SD Punjab), entre 9 e 24 meses de idade, sem sintomas e complicações [Internet]. 2024 [cited 2025 Feb 23]. Available from: https://www.gov.br/conitec/pt-br/midias/relatorios/2024/20240307_Relatrio_873_Hidroxiureia500mg_DOENAFALCIFORME.pdf

3. Brasil. Ministério da Saúde. Secretaria de Ciência, Tecnologia e Inovação e do Complexo Econômico-Industrial da Saúde - SECTICS. Relatório de Recomendação No 872 - Hidroxiureia 100 mg e 1000 mg para o tratamento de pacientes com doença falciforme com pelo menos 9 meses de idade [Internet]. 2024 [cited 2025 Feb 23]. Available from: https://www.gov.br/conitec/pt-br/midias/relatorios/2024/20240307_Relatrio_872_Hidroxiureia_100_1000_DOENAFALCIFORMEPDF.pdf

4. Brasil. Ministério da Saúde. Secretaria de Ciência, Tecnologia e Inovação e do Complexo Econômico-Industrial da Saúde - SECTICS. Relatório de Recomendação No 874 - Alfaepoetina para o tratamento de pacientes com doença falciforme apresentando declínio da função renal e piora dos níveis de hemoglobina. [Internet]. 2024 [cited 2025 Feb 23]. Available from: <https://www.gov.br/conitec/pt-br/midias/relatorios/2024/alfaepoetina-para-o-tratamento-de-pacientes-com-doenca-falciforme-apresentando-declinio-da-funcao-renal-e-piora-dos-niveis-de-hemoglobina.pdf>

**Table S2:** Summary of Finding (SoF) and GRADE EtD for technologies assessed for listing

| Question 1: Is epoetin alfa in combination with standard care compared to standard care, effective, safe, cost-effective and economically viable for the treatment of adults with sickle cell disease (SCD) who have renal impairment associated with worsening anemia? | | | |
| --- | --- | --- | --- |
| Background and motivation: One of the main complications of SCD – which occurs especially in people with the hemoglobin (Hb) SS and Hb Sbeta0 genotypes – is sickle cell nephropathy, which can progress from asymptomatic conditions to chronic kidney disease (CKD). In addition to standard treatment with hydroxyurea and blood transfusions, international guidelines recommend treatment with erythropoiesis-stimulating agents (ESAs), such as epoetin alfa, for patients with renal impairment. The use of this drug is consolidated for the treatment of anemia in CKD in patients without SCD and is listed by SUS for CKD patients. | | | |
| Patient or population: Adults with SCD, with renal impairment associated with worsening anemia  Setting: Outpatient treatment, primary care  Intervention: Epoetin alfa (recombinant human erythropoietin) + standard care  Comparison: Standard care (regular transfusions, hydroxyurea and folic acid) | | | |
| Outcomes | Impact | Nº of studies/ design | Certainty of the evidence (GRADE) |
| Hb levels | The percentage change in mean Hb concentration was 4% to 32.8% compared to baseline | 7 / observational | VERY LOW ⨁◯◯◯ |
| Regular transfusion | The three studies qualitatively reported that there was a reduction in the need for blood transfusions after the use of epoetin alfa. | 3 / observational | VERY LOW ⨁◯◯◯ |
| Hb F levels | Differences between pre- and post-treatment Hb F percentage levels ranged from 5.2% to 17.1%. | 7 / observational | VERY LOW ⨁◯◯◯ |
| Vaso-occlusive crises (VOCs) | There was no increase in VOC rates. One study reported that the frequency of VOCs was significantly reduced after the use of epoetin alfa. | 4 / observational | VERY LOW ⨁◯◯◯ |
| Venous thromboembolism (VTE) | There was no increase in VTE rates. | 2 / observational | VERY LOW ⨁◯◯◯ |
| Serious adverse events | One patient experienced liver failure probably related to the use of epoetin alfa. No other serious adverse events or other important clinical changes related to the use of epoetin alfa were reported. | 2 / observational | VERY LOW ⨁◯◯◯ |
| Explanations:  The risk of bias was considered high for observational studies, mainly due to the absence of a comparator group and the presence of confounding factors not considered in the data analysis.  Small sample size of the studies and the results of continuous outcomes demonstrated large amplitude of the minimum-maximum ranges (95% confidence intervals were not estimated) and it is not possible to estimate the optimal size of the information from the qualitative report of the dichotomous outcomes. | | | |
| Problem | SCD is a severe genetic disorder that primarily affects populations of African descent, causing chronic hemolytic anemia, pain crises, and multi-organ damage. One of the major complications of SCD is sickle cell nephropathy, which can progress to chronic kidney disease (CKD) and end-stage renal disease (ESRD). CKD in SCD patients is associated with increased morbidity and mortality, as well as a significant burden on healthcare systems due to the need for frequent transfusions, iron chelation therapy, and dialysis in advanced stages. Despite the availability of supportive treatments such as hydroxyurea, many patients with SCD and renal impairment experience worsening anemia, requiring alternative therapies like erythropoiesis-stimulating agents (ESAs) to manage Hb levels and reduce transfusion dependence. | | |
| Benefits and harm | Evidence suggests that epoetin alfa can be beneficial in the treatment of adult patients with SCD and renal impairment associated with progressive anemia. Eight before-and-after observational studies were included, in which epoetin alfa was associated with a significant increase in Hb concentration compared to baseline values (p-value <0.05). Additionally, there was an improvement in Hb F levels as well as a reduction in the need for blood transfusion.  Regarding risks, no significant increases in the occurrence of VOC or VTE were observed, suggesting a favorable safety profile. However, the quality of evidence was rated very low due to risk of bias and imprecision. This highlights the need for continuous monitoring of patients to assess long-term safety. | | |
| Resource use | The cost-effectiveness analysis used a decision tree model with a one-year time horizon. The results showed that adding epoetin alfa into standard care resulted in a modest incremental benefit of 0.033 quality-adjusted life years (QALYs) and a cost reduction of R$ 11,564 per patient. Epoetin alfa was considered a dominant alternative, as it presented lower costs and better clinical outcomes compared to the current standard of care alone. Sensitivity analyses confirmed the robustness of the results, indicating that epoetin alfa may be cost-effective in SUS. | | |
| Equity | SCD disproportionately affects vulnerable populations, especially groups with limited access to quality healthcare services. However, no equity assessments were made. | | |
| Acceptability | The acceptability of epoetin alfa treatment among patients and healthcare professionals is considered high, mainly due to the potential reduction in dependence on frequent blood transfusions and their adverse effects. However, the absence of robust evidence or international guidelines for the use of epoetin alfa in SCD patients may create uncertainty among healthcare professionals. The need for objective criteria to initiate and discontinue treatment was discussed, including defining renal function decline and sustained Hb reduction. | | |
| Feasibility | The budget impact analysis (BIA) estimated that the eligible population for receiving epoetin alfa would be an average of 5,274 patients per year, with a diffusion rate ranging from 10% in the first year to 50% in the fifth year. The direct cost of acquiring the drug ranged from R$ 806,129 in the first year to R$ 4,853,242 in the fifth year of incorporation. The BIA suggests that its incorporation into SUS is feasible and could result in significant savings for the Brazilian healthcare system. | | |
| Question 2: Is the use of hydroxyurea effective, effective and safe for the treatment of patients with SCD (Hb SS, Hb Sbeta0 and Hb SD Punjab) between 9 and 24 months of age, without symptoms or complications, when compared to standard care (folic acid, analgesics and anti-inflammatories)? | | | |
| Background and motivation: The use of hydroxyurea in children with SCD has been extensively studied, and there are specific guidelines for its use in this population recommending hydroxyurea therapy starting at 9 months of age, regardless of symptoms or complications. Currently, the Brazilian National Guidelines for SCD recommends treatment with hydroxyurea for individuals from 2 years of age and in special cases for the age group between 9 months and 2 years. When considering the use of hydroxyurea in young children with SCD, it is essential to weigh the potential benefits and risks. | | | |
| Patient or population: Patients with SCD (Hb SS, Hb Sbeta0 and Hb SD Punjab) between 9 and 24 months of age, without symptoms or complications.  Setting: Outpatient treatment  Intervention: Hydroxyurea  Comparison: Standard care (adjuvant treatment such as folic acid, analgesics and anti-inflammatories) | | | |
| Outcomes | Impact | Nº of studies/ design | Certainty of the evidence (GRADE) |
| Hb levels | MD 0.9 [95% CI: 0.5-1.3], p-value <0.001 | 1 / randomized clinical trial | MODERATE ⨁⨁⨁◯ |
| Hb F levels | MD 6.7% [95% CI: 4.8-8.7%], p-value <0.001 | 1 / randomized clinical trial | MODERATE ⨁⨁⨁◯ |
| Splenic function | MD -11% (95% CI: -26 to 5) p-value = 0.21  Absolute effect 11% lower (26 lower to 5 higher) | 1 / randomized clinical trial | MODERATE ⨁⨁⨁◯ |
| Glomerular filtration rate | MD 2% (95% CI: -16 to +20) p-value = 0.84  Absolute effect 2 mL/min/1.73m2 highest (16 lowest to 20 highest) | 1 / randomized clinical trial | MODERATE ⨁⨁⨁◯ |
| Pain | HR 0.59 [95% CI: 0.42-0.83], p-value = 0.002 | 1 / randomized clinical trial | MODERATE ⨁⨁⨁◯ |
| Acute chest syndrome | HR 0.36 [95% CI: 0.15-0.87], p-value = 0.02 | 1 / randomized clinical trial | MODERATE ⨁⨁⨁◯ |
| Transfusion requirements | HR 0.55 [95% CI: 0.32-0.96], p-value = 0.03 | 1 / randomized clinical trial | MODERATE ⨁⨁⨁◯ |
| Hospitalizations | HR 0.73 [95% CI: 0.53-1.00], p-value = 0.05 | 1 / randomized clinical trial | MODERATE ⨁⨁⨁◯ |
| Explanations:  Optimal information size (119 to 574 participants per group) not reached for the outcomes.  95% CI: 95% Confidence interval; HR: Hazard ratio; MD: Mean difference | | | |
| Problem | SCD is a severe genetic disorder that primarily affects populations of African descent and is associated with high morbidity and mortality, particularly in children. The condition leads to chronic hemolytic anemia, painful VOCs, and multi-organ damage. Early intervention with hydroxyurea in infants between 9 and 24 months of age has been proposed as a strategy to mitigate disease progression, reduce complications, and improve long-term outcomes. This evaluation aims to determine the benefits, harms, cost-effectiveness, equity, acceptability, and feasibility of expanding hydroxyurea eligibility criteria related to age into the treatment protocol within the Brazilian Unified Health System (SUS). | | |
| Benefits and harm | Hydroxyurea has demonstrated significant clinical benefits for young children with SCD, as evidenced by the BABY HUG randomized controlled trial. Compared to placebo, hydroxyurea improved Hb levels by an average of 0.9 g/dL (95% CI: 0.5-1.3; p-value <0.001) and increased fetal Hb by 6.7% (95% CI: 4.8-8.7%; p-value <0.001). It also reduced the incidence of VOCs (HR: 0.59; 95% CI: 0.42-0.83; p-value = 0.002), acute chest syndrome (HR 0.36; 95% CI: 0.15-0.87; p-value = 0.02), and transfusion rates (HR 0.55; 95% CI: 0.32-0.96; p-value = 0.03). No significant increase in hospitalization rates or severe adverse events was observed. The overall confidence in the evidence was rated as moderate. | | |
| Resource use | The cost-effectiveness analysis showed that hydroxyurea provides greater clinical benefits but also results in higher treatment costs compared to standard care (folic acid, analgesics, and anti-inflammatories). The incremental cost-effectiveness ratio (ICER) was estimated at R$ 232.26 per VOC avoided and R$ 12,257.73 per QALY gained. Sensitivity analyses indicated that hydroxyurea remains cost-effective under various scenarios and is within the willingness-to-pay threshold established by CONITEC. | | |
| Equity | SCD disproportionately affects marginalized populations with limited access to healthcare. Early treatment with hydroxyurea can reduce disease severity and improve long-term health outcomes, particularly in underserved communities. However, no equity assessments were made. | | |
| Acceptability | Hydroxyurea is well-accepted among healthcare providers and caregivers, particularly due to its proven efficacy in reducing painful crises and hospitalizations. However, concerns remain regarding the lack of pediatric-friendly formulations and potential barriers to adherence, such as caregiver education and regular monitoring requirements. Public consultation findings indicated broad support for the incorporation of hydroxyurea in SUS for children aged 9 to 24 months, with stakeholders highlighting its positive impact on disease management and quality of life. | | |
| Feasibility | The BIA estimated that the eligible patient population would range from 275 to 461 children annually. The projected cumulative budget impact over five years varied from R$ 105,556 to R$ 484,805, depending on the diffusion rate and population estimates. Despite the increase in direct costs, long-term savings from reduced hospitalizations and complications are expected to offset expenses, supporting the feasibility of listing hydroxyurea in SUS. | | |
| Question 3: Is the use of hydroxyurea 100 mg and 1000 mg economically viable for the treatment of patients with SCD over 9 months of age? | | | |
| Background and motivation: The objective was to analyze the budgetary impact and viability of a possible incorporation of hydroxyurea in the concentrations of 100 and 1000 mg for the treatment of individuals aged at least 9 months | | | |
| Patient or population: Patients with SCD who are at least 9 months old.  Setting:  Intervention: Hydroxyurea in concentrations of 100 mg and 1000 mg.  Comparison: | | | |
| Evidence synthesis: It was considered that the new formulations (100 mg and 1000 mg) are bioequivalent or similar to the 500 mg formulation, and, for this reason, only an economic viability assessment was made. Certainty of the evidence was not assessed since the studies were based on bioequivalence analyses. | | | |
| Problem | Hydroxyurea has been widely recognized as an effective therapy for SCD, and expanding its availability in different formulations (100 mg and 1000 mg) aims to improve treatment adherence and dose flexibility and adjustment, especially for kids. The listing of these formulations in SUS seeks to enhance treatment accessibility and optimize patient outcomes. | | |
| Benefits and harm | Evidence suggests that the use of hydroxyurea in 100 mg and 1000 mg formulations maintains efficacy comparable to the currently available 500 mg capsules. | | |
| Resource use | No economic evaluation was performed, since this medication is already available in SUS as a 500 mg capsule presentation, and it is understood that it is similar in terms of clinical efficacy in relation to the 100 and 1000 mg concentrations. | | |
| Equity | Not discussed or assessed. | | |
| Acceptability | The acceptability of hydroxyurea in 100 and 1000 mg formulations is expected to be high among both healthcare professionals and patients. Public consultation findings highlighted that caregivers and physicians welcomed the additional dosing options, particularly for pediatric patients who previously required the manipulation of 500 mg capsules to achieve appropriate dosing. Despite broad acceptance, concerns were raised regarding the availability and potential logistical challenges of distribution within SUS. | | |
| Feasibility | The BIA estimated that incorporating hydroxyurea 100 mg and 1000 mg in SUS would lead to an accumulated five-year cost of approximately R$ 396.7 million for patients aged nine months and older. The projected eligible population includes approximately 17,400 individuals per year, with a market penetration ranging from 10-50% over the implementation period. | | |

* After announcement of the cancellation of the marketing authorization, the research question of crizanlizumab was not discussed for recommendation, and the report did not advance for evaluation by CONITEC.

**Figure S1:** Diagnostic workflow and referral to Reference Centers.


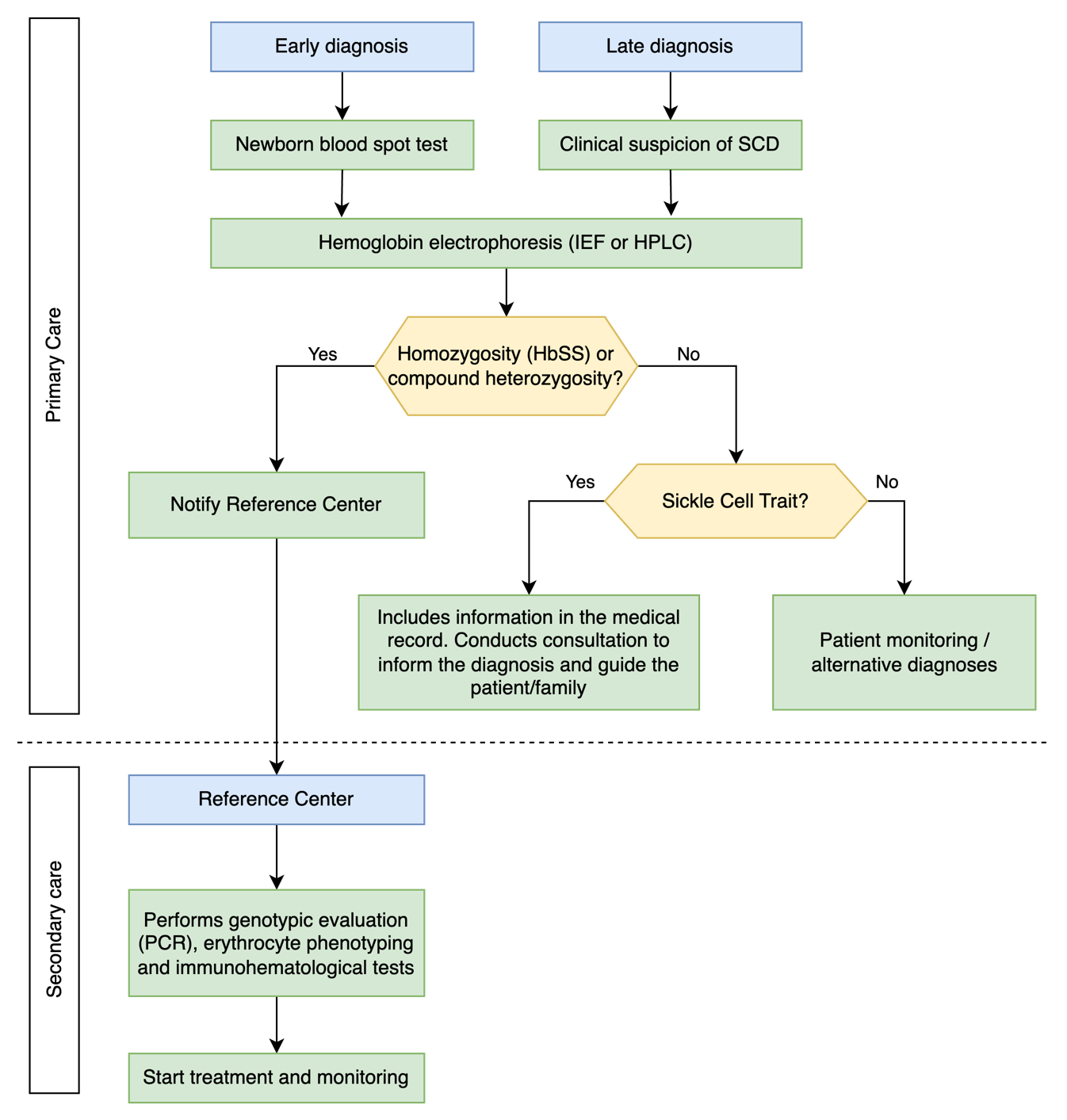


**Table S3:** Inclusion and exclusion criteria for using treatments listed in the protocol

| Technology / treatment | Inclusion/exclusion criteria |
| --- | --- |
| Hydroxyurea | The patient must meet the following criteria:   - Hb fractionation test by high-performance liquid chromatography (HPLC), Hb electrophoresis, isoelectric focusing (IEF), or molecular testing consistent with SCD types Hb SS, Hb Sβ⁰, severe Hb Sβ⁺, and Hb SD Punjab, and age 9 months or older; or - Hb fractionation test by HPLC, Hb electrophoresis, IEF, or molecular testing consistent with SCD types Hb SC, Hb SD, or Hb Sβ-thalassemia, and age 2 years or older.   Additionally, treatment-naïve patients presenting any of the following conditions will be excluded from hydroxyurea treatment:   - Neutrophil count below 1,500/mm³ for patients over one year old, or 1,000/mm³ for patients under one year old; - Hb below 4.5 g/dL; - Reticulocyte count below 80,000/mm³ (when Hb is less than 8 g/dL); - Platelet count below 80,000/mm³; - Pregnant women or sexually active women not using contraceptive methods; or - Active liver disease (HBV or HCV infection), unless deemed contraindicated by the prescriber or hepatic function cannot be monitored.   Children with SCD types Hb SC, Hb SD, or Hb Sβ-thalassemia under 2 years of age should be carefully evaluated for using hydroxyurea, considering risks and benefits. In these cases, the medication may be prescribed starting at 9 months of age, using the same inclusion criteria, with the addition of at least one of the following factors:   - Dactylitis (in the first year of life); or - Hb concentration less than 7 g/dL (average of three values outside of acute events); or - White blood cell count greater than 20,000/mm³ (average of three values outside of acute events). |
| Special cases | Hydroxyurea therapy in children under 2 years with Hb SC, Hb SD, or Hb Sβ-thalassemia SCD may be prescribed from 9 months of age if inclusion criteria are met, along with at least one of the following:   - Dactylitis (in the first year); - Hb <7 g/dL (average of 3 values outside acute events); - White blood cell count >20,000/mm³ (average of 3 values outside acute events). |
| Epoetin alfa | The patient must be 18 years or older and meet one of the following criteria:   - Hb fractionation test by HPLC, Hb electrophoresis, IEF, or molecular testing consistent with SCD types Hb SS or Hb Sβ⁰, be undergoing hydroxyurea treatment, and require more than three red blood cell (RBC) transfusions per year to maintain a Hb level equal to or above 8.5 g/dL, or present a reduction of 1.5 g/dL or more in baseline Hb; or - Hb fractionation test by HPLC, Hb electrophoresis, IEF, or molecular testing consistent with SCD type Hb SS or Hb Sβ⁰ with hyperhemolysis syndrome. |
| Penicillin V (Phenoxymethylpenicillin) | The patient must be 5 years old or younger. |
| Hematopoietic Stem Cell Transplantation (HSCT) | For allogeneic HSCT from an HLA-identical or haploidentical related donor, using umbilical cord blood, peripheral blood, or bone marrow, the patient must meet all the following inclusion criteria:   - Hb electrophoresis or molecular testing consistent with SCD types Hb SS or Hb Sβ-thalassemia; - Undergoing hydroxyurea treatment; - Present at least one of the following conditions: - Neurological alteration due to a cerebrovascular accident, with neurological impairment lasting more than 24 hours or imaging abnormalities; - Cerebrovascular disease associated with SCD; - More than two severe VOCs (including acute chest syndrome) within the last year; - More than one episode of priapism; - Presence of more than two antibodies in patients under hyper-transfusion, or one high-frequency antibody; - Osteonecrosis in more than one joint.   Patients with comorbidities compromising transplant outcomes, as assessed and defined by the transplant team, will be excluded from HSCT. |

**Table S4:** Recommended tests and monitoring frequency for patients receiving hydroxyurea

| **Test** | **Initial evaluation** | **Monitoring (recommended for dose adjustment)** |
| --- | --- | --- |
| Hb electrophoresis | ✓ | Not recommended |
| Hb F measurement | ✓ | Every 8-12 weeks until the maintenance dose is reached; then every 6 months. |
| Complete blood count (CBC) with platelet count, reticulocyte count, and mean corpuscular volume (MCV) assessment | ✓ | Every 2 weeks until the maintenance dose is reached; then every 12 weeks. |
| Serology for hepatitis B, hepatitis C, and HIV | ✓ | Not recommended |
| Serum creatinine, AST (TGO), and ALT (TGP) levels | ✓ | Every 4 weeks until the maintenance dose is reached; then every 12 weeks. |
| Serum β-HCG measurement or rapid pregnancy test | ✓ |  |

**Table S5:** Hematological parameters for toxicity assessment and hydroxyurea dose adjustment.

| **Parameter** | **Acceptable Level** | **Toxic Level** |
| --- | --- | --- |
| Neutrophils (cells/mm³) | Greater than 2,000* | Less than 1,500 |
| Platelets (cells/mm³) | Greater than 85,000 | Less than 80,000 |
| Hb (g/dL) | Greater than 5.3 | Less than 4.5 |
| Reticulocytes (cells/mm³) * | Greater than 95,000 | Less than 80,000 |

Note: Reticulocyte count is required until Hb reaches a level greater than 9 g/dL.

The absolute neutrophil count (ANC) target recommended in the literature is 2,000 cells/mm³; however, younger individuals with lower baseline counts may safely tolerate ANC levels as low as 1,250 cells/mm³. For children under 1 year of age, acceptable levels are greater than 1,000 cells/mm³, and for those over 1 year, levels should be greater than 1,500 cells/mm³.

Figure S2. Timeline for transition process


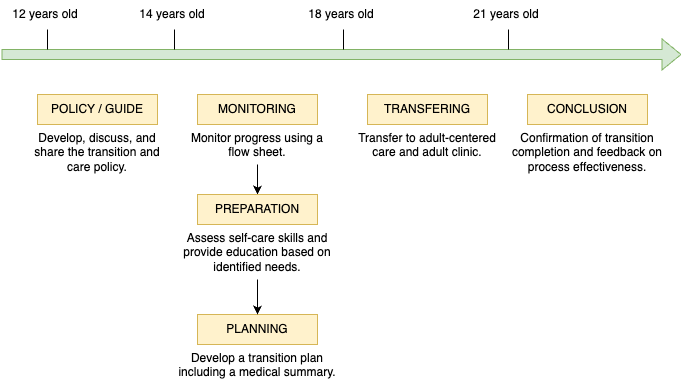

Supplement: Supplementary file 1 [file mmc1.docx]
